# Interpreting blood–brain barrier bypass claims in reperfused stroke: a minimum reporting framework for intracalvarial immune-assisted nanoparticle delivery

**DOI:** 10.3389/fneur.2026.1818232

**Published:** 2026-06-19

**Authors:** Dandan Liu, Hezhong Ouyang, Xiaojin Wei, Zhengwei Chen, Shiyao Zhang, Fuling Yan

**Affiliations:** 1 Department of Neurology, The People's Hospital of Danyang, Affiliated Danyang Hospital of Nantong University, Zhenjiang, Jiangsu, China; 2 Department of Neurology, Affiliated ZhongDa Hospital, School of Medicine, Southeast University, Nanjing, Jiangsu, China; 3 Department of Neurology, Second Affiliated Hospital of Xuzhou Medical University, Xuzhou, Jiangsu, China

**Keywords:** blood-brain barrier, calvarial immunity, drug delivery, nanoparticle (NP), stroke

## Introduction

Discoveries of skull-associated immunity and skull–meningeal vascular channels have changed how neuroimmune trafficking is understood. These channels connect calvarial marrow and meningeal or brain-border compartments, and calvarial marrow can serve as a local myeloid reservoir for central nervous system (CNS) inflammation (1–3). Intracalvarial or intracalvariosseous delivery has therefore attracted interest as a potential route for CNS drug delivery in stroke (4, 5).

Intracalvarial or intracalvariosseous delivery typically refers to administration into the skull bone marrow or diploic space, with the aim of engaging local immune-cell populations and skull-meningeal vascular channels. In this route hypothesis, nanoparticles may be taken up by resident or recruited myeloid cells, which can traffic through skull-meningeal interfaces toward meningeal or brain-border compartments.

Importantly, this pathway is expected to preferentially support transport to border-associated or immune-cell-accessible compartments, rather than direct, unrestricted entry into the brain parenchyma. Therefore, although this route provides a biologically plausible mechanism for CNS delivery, it does not by itself establish parenchymal blood–brain barrier (BBB) bypass without additional evidence of compartment-resolved localization and timing-aligned BBB integrity.

In reperfused stroke, however, BBB integrity changes during the same period in which delivery and sampling are often performed (6–11). Increased nanoparticle (NP) signal in the injured hemisphere therefore does not, by itself, establish strict BBB bypass. The signal may reflect skull-assisted immune transport, injury-enabled BBB permeability, vascular or perivascular retention, lesion-driven immune recruitment, or a combination of these mechanisms (12–14).

This Opinion proposes a conservative interpretation and reporting framework for studies of intracalvarial immune-assisted NP delivery in reperfused stroke. The goal is not to dismiss the emerging strategy, but to define the minimum evidence needed before strong BBB-bypass language is used. This framework provides a practical, testable structure rather than a purely conceptual viewpoint.

## Why mechanistic language matters in reperfused stroke

BBB dysfunction after ischemia–reperfusion is not static. Experimental and clinical studies show that permeability and its assessment depend on timing, reperfusion status, imaging or tracer method, and tissue compartment (6–11). Early changes may include enhanced transcellular transport, whereas later injury can involve tight-junction disruption and paracellular leakage (6, 7). Thus, a post-stroke increase in hemispheric NP signal should first be interpreted as a tissue-associated injury signal rather than proof of an anatomically independent route across, or around, the BBB.

Three non-exclusive mechanisms should be considered. First, skull-assisted immune transport may deliver NP-loaded immune cells from calvarial marrow or meningeal interfaces toward the injured brain. Second, injury-enabled access may occur when ischemia–reperfusion alters BBB permeability during the delivery window. Third, lesion-driven immune recruitment may increase local immune-cell density and NP signal at the site of injury. The temporal and compartmental dynamics of post-stroke immune-cell accumulation support this need for caution (12–14). Without separating these mechanisms, the phrase “BBB bypass” risks overinterpreting a biologically plausible but incompletely localized signal.

## Terminology framework for tissue-associated signal

To reduce ambiguity, we recommend separating three terms. “Skull-assisted immune transport” should be reserved for the route hypothesis: immune cells or other carriers associated with skull marrow, skull–meningeal channels, or meningeal interfaces contribute to NP movement toward CNS-border or injured-brain compartments. “Injury-enabled access” should describe accumulation facilitated by BBB disruption, vascular leakage, reperfusion injury, or inflammatory permeability. “BBB bypass” should be used only when the data show delivery to the intended parenchymal compartment without relying on concurrent BBB opening.

Similarly, “brain signal” should be defined operationally. At minimum, authors should state whether the measured signal is intravascular, perivascular, border-compartment-associated, or true extravascular parenchymal signal. This distinction is essential in reperfused stroke because each compartment may produce a hemispheric signal while implying different delivery mechanisms.

## What current studies suggest and what they do not yet establish

Recent studies, including Liu et al. and Gao et al. (4, 5), provide important evidence supporting the plausibility of skull-related access routes and calvarial immune-cell participation, with reported improvements in therapeutic outcomes. However, under stricter mechanistic interpretation, such findings do not by themselves establish definitive BBB bypass. In particular, key criteria remain incompletely resolved: whether BBB integrity was assessed at time points aligned with dosing and sampling, whether the detected signal was localized beyond vascular and perivascular compartments to true extravascular parenchymal entry, and whether absolute brain exposure was increased under dose- and time-matched conditions rather than inferred from organ ratios. A strong BBB-bypass claim therefore requires explicit resolution of these issues, beyond increased injured-hemisphere signal or therapeutic effect alone.

Thus, the key interpretive question is not whether intracalvarial immune-assisted delivery is promising; it is. The key question is what level of evidence is sufficient to support specific mechanistic language.

## A minimum reporting framework

We propose four minimum domains that should be reported before strict BBB-bypass terminology is used.

First, timing alignment is essential. Authors should report ischemia onset, reperfusion, dosing, BBB assessment, and tissue sampling on the same time axis. BBB readouts should be aligned with the delivery window rather than measured only at a distant or convenient time point. This recommendation is supported by BBB-imaging and experimental literature showing that barrier status varies with time and reperfusion context (6–11).

Second, compartment-resolved localization should be required. Useful controls may include vascular perfusion at sacrifice, intravascular labeling before tissue collection, vascular marker co-staining, distance-to-vessel analysis, meningeal whole-mount imaging, optical clearing, or three-dimensional reconstruction. The objective is to distinguish extravascular parenchymal signal from signal in vessels, perivascular spaces, leptomeninges, skull channels, or other border compartments. Post-stroke neutrophil and myeloid-cell studies illustrate why this separation is necessary (12–14).

Third, exposure context should be reported. Absolute NP concentration in blood or plasma, liver, spleen, lung, kidney, contralateral brain, and injured brain should be provided when feasible. Ratios alone are insufficient. For example, a brain-to-liver ratio can rise because liver exposure falls, even if absolute injured-brain exposure is unchanged. This recommendation is consistent with broader bio-nano reporting standards and nanoparticle-delivery analyses emphasizing quantitative exposure, protocol details, and reproducibility (15, 16).

Fourth, statistics should match the experimental design. Multiple sections, fields, cells, or particles from the same animal are nested observations, not independent biological replicates. The animal should usually be the unit of inference, or models should explicitly account for nesting. This recommendation aligns with guidance on dependency, pseudoreplication, and transparent *in vivo* reporting (17–19).

**Figure 1:**
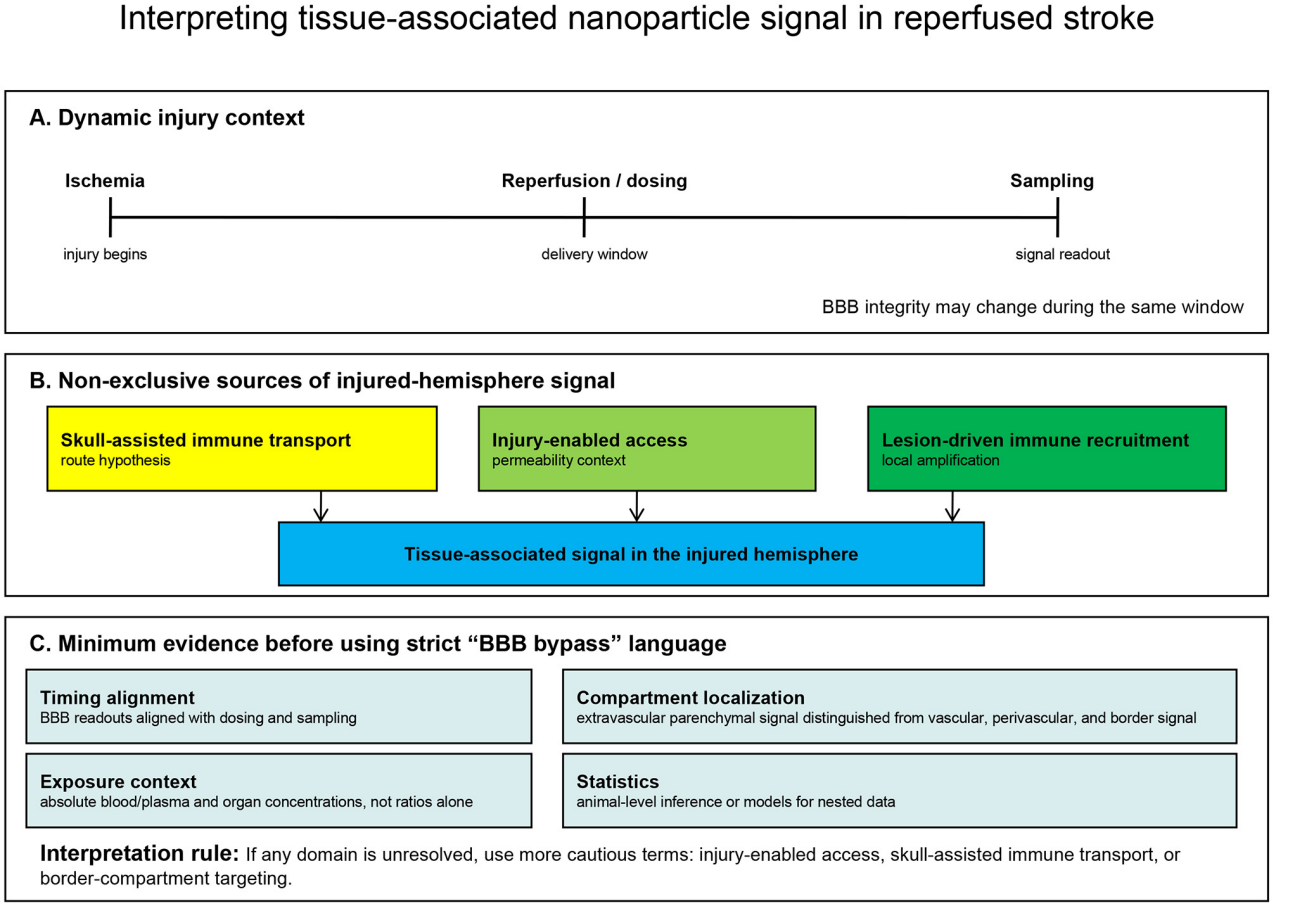
Conceptual framework and compact reporting checklist for interpreting tissue-associated nanoparticle (NP) signal in reperfused stroke. **(A)** After ischemia–reperfusion, blood–brain barrier (BBB) status may change across the dosing and sampling window. **(B)** Signal detected in the injured hemisphere may reflect non-exclusive mechanisms, including skull-assisted immune transport, injury-enabled permeability-mediated access, and lesion-driven immune recruitment. **(C)** A strict BBB-bypass interpretation requires timing-aligned BBB assessment, compartment-resolved localization demonstrating extravascular parenchymal entry, dose- and time-matched exposure context, and analysis that respects biological independence. If any domain remains unresolved, more cautious terms such as injury-enabled access, skull-assisted immune transport, or border-compartment targeting should be used. The checklist provides a stepwise operational guide for reporting and interpretation.

These domains are summarized in Figure 1, which integrates the conceptual interpretation logic with a compact reporting checklist to facilitate practical implementation and rapid assessment by readers.

## Translational implications

These standards are relevant to clinical translation. If early human imaging or pharmacokinetic studies show lesion-associated accumulation after reperfusion, the signal should not automatically be interpreted as BBB bypass. Clinical BBB-assessment studies show that early barrier disruption can occur after focal ischemia and can be associated with reperfusion and hemorrhagic transformation risk (10, 11). Therefore, clinical reports should specify timing from onset and reperfusion, imaging modality, vascular signal controls, and whether the signal is compatible with vascular, perivascular, meningeal, or parenchymal localization. As intracalvariosseous approaches move into prospective clinical-trial design, conservative terminology becomes increasingly important (20).

Using cautious language does not weaken the translational value of intracalvarial or intracalvariosseous delivery. Instead, it protects the field from premature mechanistic conclusions and makes positive findings more reproducible and clinically interpretable. By anchoring each recommendation to existing experimental and methodological literature, this framework addresses the concern that interpretation of BBB bypass has previously lacked evidentiary grounding.

## Limitations

This framework has limitations. It is a conceptual reporting framework, not a validated experimental protocol. Some recommendations, such as three-dimensional localization or detailed biodistribution, may be difficult to implement in every laboratory. The framework is also focused on reperfused stroke, where BBB status and immune recruitment are especially dynamic. Other CNS diseases may require modified criteria.

## Conclusion

Intracalvarial immune-assisted NP delivery is a promising strategy for CNS drug delivery, but reperfused stroke creates a complex interpretive setting. Increased injured-hemisphere signal should be reported as a tissue-associated signal unless timing-aligned BBB assessment, compartment localization, exposure context, and animal-level statistics support stronger language. A shared reporting framework can help the field distinguish route innovation from injury-enabled accumulation and can make future preclinical and translational studies easier to compare.
